# Prevalence of frailty and pre-frailty and related factors in older adults with cardio-cerebral vascular disease in China: a national cross-sectional study

**DOI:** 10.3389/fpubh.2023.1168792

**Published:** 2023-06-16

**Authors:** Xue-zhai Zeng, Ling-bing Meng, Na Jia, Jing Shi, Chi Zhang, Ying-ying Li, Xing Hu, Jia-bin Hu, Jian-yi Li, Di-shan Wu, Hui Li, Xin Qi, Hua Wang, Qiu-xia Zhang, Juan Li, De-ping Liu

**Affiliations:** ^1^Department of Cardiology, Beijing Hospital, National Center of Gerontology, Institute of Geriatric Medicine, Chinese Academy of Medical Sciences, Beijing, China; ^2^Department of Geriatrics, Beijing Hospital, National Center of Gerontology, Institute of Geriatric Medicine, Chinese Academy of Medical Sciences, Beijing, China; ^3^The Key Laboratory of Geriatrics, Beijing Institute of Geriatrics, Institute of Geriatric Medicine, Chinese Academy of Medical Sciences, Beijing Hospital/National Center of Gerontology of National Health Commission, Beijing, China; ^4^Health Service Department of the Guard Bureau of the Joint Staff Department, Beijing, China; ^5^China Research Center on Ageing, Beijing, China; ^6^Institute of Psychology, Chinese Academy of Sciences, Beijing, China

**Keywords:** cardio-cerebral vascular disease, China, frailty, older adults, pre-frailty, prevalence

## Abstract

**Objective:**

Frailty increases adverse clinical outcomes in older patients with cardio-cerebral vascular disease (CCVD). The aim of this study was to investigate the prevalence of frailty and pre-frailty in older adults with CCVD in China and the factors associated with it.

**Research design and methods:**

In this cross-sectional study, we used data from the fourth Sample Survey of Aged Population in Urban and Rural China. We used the frailty index for frailty and pre-frailty assessment, and the diagnosis of CCVD in older adults was self-reported.

**Results:**

A total of 53,668 older patients with CCVD were enrolled in the study. The age-standardized prevalence of frailty and pre-frailty in older patients with CCVD was 22.6% (95% CI 22.3–23.0%) and 60.1% (95% CI 59.7–60.5%). Multinomial logistic regression analyses showed that being female, increasing age, rural residence, illiteracy, widowhood, ethnic minority, living alone, no health screening during the last year, hospitalization during the last year, difficult financial status, comorbid chronic conditions, and disability in activities of daily living were associated with frailty and pre-frailty in older patients with CCVD.

**Conclusion:**

CCVD is strongly associated with frailty and pre-frailty in older Chinese people, and assessment of frailty should become routine in the management of older CCVD patients. Appropriate public health prevention strategies should be developed based on identified risk factors for frailty in older CCVD patients, which can help prevent, ameliorate or reverse the development of frailty in CCVD in the older population.

## Introduction

The seventh national population census in China conducted in 2020 showed that the population aged 60 or above was 264.02 million, representing 18.70%, up 5.44 percentage points from 2010, indicating that China has entered an aging society and the rate of aging is accelerating (1). With the advent of an aging population, the prevalence of cardio-cerebral vascular disease (CCVD) in China is on the rise, and the Cardiovascular Health and Diseases Report in China 2021 estimates that 330 million people have CCVD, and mortality from CCVD is the number one cause of death in both urban and rural areas, at 46.74% in rural areas and 44.26% in urban areas, it is higher than tumors and other diseases (2). CCVD imposes a heavy economic burden on the older adults and society, and has become a major public health problem. Frailty is a common geriatric syndrome, a clinical state that reflects heterogeneity between biological and chronological age due to a decrease in the physiological reserve and function of multiple systems resulting in a diminished ability of the organism to maintain homeostasis, an increased vulnerability of the organism and a diminished resistance to stress (3–6). Studies have shown a weighted prevalence of 11% (range 4–59%) for frailty in community-dwelling older adults (7). Frail older adults are at significantly increased risk for falls, disability, hospitalization, and death, and increase the burden on societal health care resources (3, 8, 9). Studies have shown that the prevalence of frailty in older adults with CCVD ranges from 10 to 60%, with a significant increase in adverse outcome events in older adults with frailty compared to those without frailty (10–13). In clinical practice, frail patients with CCVD require an individualized approach and optimized treatment decisions (14, 15). Early recognition of frailty is crucial to prevent the development of disability, dependence on others and a reduced quality of life (13). In order to reduce the burden of frailty in older patients with CCVD, corresponding public health policies are urgently needed, but the study of frailty in China started late, and there are few studies on the prevalence of frailty in older patients with CCVD. Our study aimed to analyze the prevalence of frailty and pre-frailty and related factors in older patients with CCVD in China, using data from the 4th Sample Survey of Aged Population in Urban and Rural China (SSAPUR) in 2015. The results of our study can provide valuable information for the prevention and treatment of frailty in older patients with CCVD.

## Methods

### Study design and participants

**Figure 1:**
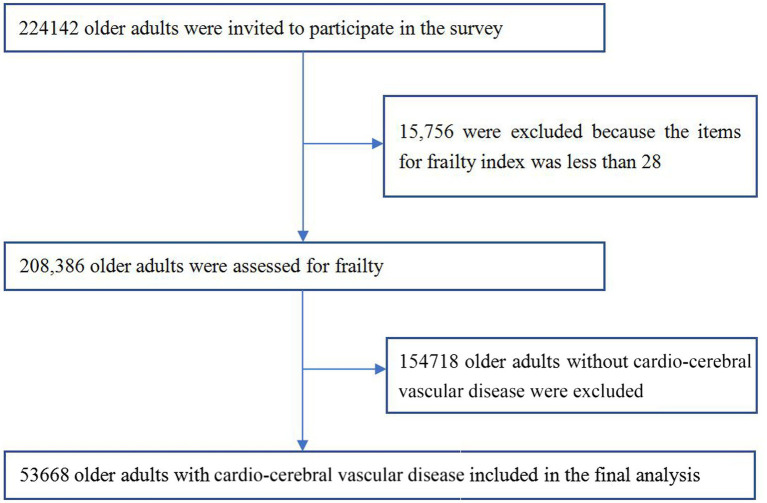
Flowchart of study participants on frailty and pre-frailty prevalence in older adults with cardio-cerebral vascular disease in China.

In this cross-sectional study, we used data from the fourth SSAPUR, in which a total of 224,142 older adults aged 60 years or older from 31 provinces/municipalities/autonomous regions in mainland China participated from 1 August 2015 to 31 August 2015. The survey used a comprehensive sampling method with stratified, multi-stage, size-proportional probability sampling and an equal-probability sampling design at the final stage to ensure the representativeness of the national older population. The sample size was allocated according to the proportion of the older population in each provinces/municipalities/autonomous regions in mainland China, and 462 counties were finally selected. From each county, four towns were selected and from each town, four communities were selected. Finally, 30 older people were selected from each community using an equidistant sampling method. The data collected through household interviews and questionnaires provided valuable insights into the living conditions of older people. More detailed information on the survey design and sampling methods can be found in previous studies (16–18). We use the frailty index (FI) to assess the frailty status of older adults. Exclusion criteria for this study: 15,756 participants out of 224,142 older adults who participated in the fourth SSAPUR were excluded because less than 28/33 items were used to construct the FI, and 154,718 older adults without a history of CCVD were excluded out of the remaining 208,386 older adults. Inclusion criteria for this study: older adults with self-reported CCVD and ≥ 28/33 items used to construct FI, a total of 53,668 older patients with CCVD were enrolled in this study (Figure 1). The study protocol was approved by the National Bureau of Statistics (No. [2014] 87) and the Beijing Hospital Ethics Committee (2021BJYYEC-294-01). Written informed consent was provided by all participants.

### Demographics

Demographic characteristics: age, sex, education, marital status, ethnicity, urban/rural, living alone, health screening during the last year, hospitalization during the last year, financial status, ease of reimbursement of medical expenses, disability in activities of daily living (ADL), co-morbid chronic diseases.

### Identification and assignment of health deficit variables for FI

We selected 33 items from the questionnaire (at least 28/33 items per subject) to construct the FI according to the FI construct consensus (19). The exact construction method has been described in our previous study (16). We classified the FI into 3 levels: robust (FI < 0.12), pre-frailty (FI ≥ 0.12 and < 0.25), and frailty (FI ≥ 0.25).

### Statistical analysis

The literature reports a 10–60% prevalence of frailty in patients with CCVD, and we used a 10% prevalence to calculate the sample size (10, 11, 13). We used PASS software (NCSS, Kaysville, UT, USA) to calculate the sample size and applied a design effect of 3 to account for a multi-stage cluster sampling design. The sample size was 10,671. Thus, our sample of over 50,000 participants in this study was adequate. Statistical analysis was performed using SPSS 24.0 (IBM Corporation, Armonk, NY, USA). Missing data were interpolated using the Markov Chain Monte Carlo (MCMC) multiple fill method. Age-adjusted prevalence of frailty and pre-frailty among older Chinese people with CCVD was calculated according to the weights established in our study. The significance of differences was assessed by Student’s t-test for continuous variables and by χ^2^-test for categorical variables. The Cochran-Armitage test was used to test for trends in the prevalence of covariates. Multinomial regression analyses were used to identify factors which were associated with frailty and pre-frailty, including age group, gender, ethnicity, place of residence, education level, marital status, living alone, health screening during the last year, hospitalization during the last year, financial status, ease of medical reimbursement, comorbid chronic conditions, and ADL disability. The level of statistical significance was defined as *p* less than 0.05.

## Results

A total of 53,668 self-reported patients with CCVD were included in our study, and the self-reported prevalence of CCVD among older adults was 25.8%, with a prevalence of 23.0% among men and 28.3% among women. The demographics and frailty risk factors by gender, urban/rural and ethnicity of older adults with CCVD are shown in Table 1.

**Table 1:** Demographics of the Chinese adults aged 60 years or older with CCVD by gender, urban/rural, and ethnic groups in 2015.

	Total (*n* = 53,668)	Men (*n* = 22,856)	Women (*n* = 30,812)	*p* value	Urban (*n* = 29,357)	Rural (*n* = 24,311)	*p* for difference	Han Chinese (*n* = 51,048)	Ethnic minorities (*n* = 2,620)	*p* for difference
Proportional of participants	100%	42.6%	57.4%	<0.001	54.7%	45.3%	<0.001	95.1%	4.9%	<0.001
Age (years)	70.9 ± 7.8	70.8 ± 7.7	70.9 ± 7.9	0.328	71.4 ± 8.0	70.2 ± 7.6	<0.001	70.9 ± 7.8	70.1 ± 7.4	<0.001
Age group				<0.001			<0.001			<0.001
60–64	26.2%	25.9%^a^	26.4%^a^		24.2%^a^	28.5%^b^		26.0%^a^	28.7%^b^	
65–69	23.2%	23.4%^a^	23.1%^a^		22.2%^a^	24.5%^b^		23.2%^a^	24.4%^a^	
70–74	18.8%	19.0%^a^	18.6%^a^		18.7%^a^	18.9%^a^		18.7%^a^	19.2%^a^	
75–79	15.6%	16.1%^a^	15.3%^b^		16.6%^a^	14.4%^b^		15.7%^a^	14.5%^a^	
80–84	10.5%	10.3%^a^	10.6%^a^		11.9%^a^	8.8%^b^		10.6%^a^	8.8%^b^	
≥85	5.7%	5.3%^a^	6.0%^b^		6.4%^a^	4.9%^b^		5.8%^a^	4.4%^b^	
Urban or rural area (Urban)	54.7%	54.7%	54.7%	0.868				55.1%	47.6%	<0.001
Education				<0.001			<0.001			<0.001
Illiterate	30.5%	13.7%^a^	42.9%^b^		24.2%^a^	28.5%^b^		26.0%^a^	28.7%^b^	
Primary school	39.7%	43.9%^a^	36.6%^b^		22.2%^a^	24.5%^b^		23.2%^a^	24.4%^a^	
Junior secondary school	18.8%	26.2%^a^	13.3%^b^		18.7%^a^	18.9%^a^		18.7%^a^	19.2%^a^	
Senior secondary school	7.5%	10.6%^a^	5.3%^b^		16.6%^a^	14.4%^b^		15.7%^a^	14.5%^a^	
Tertiary	2.2%	3.5%^a^	1.2%^b^		11.9%^a^	8.8%^b^		10.6%^a^	8.8%^b^	
University or above	1.3%	2.1%^a^	0.7%^b^		6.4%^a^	4.9%^b^		5.8%^a^	4.4%^b^	
Marital status				<0.001			<0.001			0.002
Married	70.2%	81.8%^a^	61.6%^b^		70.5%^a^	69.8%^a^		70.4%^a^	66.9%^b^	
Widowed	28.1%	15.2%^a^	37.6%^b^		27.9%^a^	28.4%^a^		27.9%^a^	31.1%^b^	
Divorced	0.8%	1.1%^a^	0.6%^b^		1.1%^a^	0.5%^b^		0.8%^a^	1.1%^a^	
Unmarried	0.9%	1.9%^a^	0.2%^b^		0.5%^a^	1.3%^b^		0.9%^a^	0.9%^a^	
Ethnicity (Non-Han)	4.9%	4.8%	5.0%	0.377	4.2%	5.6%	<0.001			
Living alone	14.2%	10.6%	16.8%	<0.001	13.5%	15.0%	<0.001	14.4%	9.1%	<0.001
Health checkup within 1 year	61.3%	62.0%	60.7%	0.002	61.6%	60.8%	0.057	61.6%	55.6%	<0.001
Hospitalized within 1 year	40.4%	41.7%	39.4%	<0.001	40.0%	40.8%	0.081	40.2%	43.7%	<0.001
Economic status				0.002			<0.001			<0.001
Very rich	1.2%	1.4%^a^	1.1%^b^		1.6%^a^	0.7%^b^		1.2%^a^	1.3%^a^	
Rich	14.5%	14.9%^a^	14.1%^b^		17.4%^a^	10.9%^b^		14.6%^a^	12.0%^b^	
Adequate	57.9%	57.7%^a^	58.1%^a^		60.5%^a^	54.8%^b^		58.0%^a^	56.3%^a^	
Poor	22.0%	21.6%^a^	22.2%^a^		17.2%^a^	27.8%^b^		21.8%^a^	25.4%^b^	
Very poor	4.4%	4.4%^a^	4.5%^a^		3.3%^a^	5.8%^b^		4.4%^a^	5.0%^a^	
Medicare				0.883						
No	0.9%	0.9%	0.9%		0.8%	1.0%	0.054	0.9%	1.3%	0.062
Convenience of medical cost reimbursement				0.004			<0.001			0.774
Highly convenient	32.0%	32.0%^a^	31.9%^a^		33.4%^a^	30.2%^b^		31.9%	32.9%	
Convenient	43.9%	44.1%^a^	43.7%^a^		43.9%^a^	43.9%^a^		43.9%	42.8%	
Less convenient	17.9%	17.3%^a^	18.4%^b^		16.9%^a^	19.1%^b^		17.9%	18.0%	
Inconvenient	4.3%	4.5%^a^	4.1%^b^		3.8%^a^	4.9%^b^		4.3%	4.2%	
Highly inconvenient	1.9%	2.1%^a^	1.9%^a^		1.9%^a^	2.0%^a^		1.9%	2.1%	
Comorbidities (≥1)	84.7%	82.3%	86.5%	<0.001	85.6%	83.6%	<0.001	84.6%	86.1%	0.037
ADL disability	8.5%	8.3%	8.7%	<0.057	8.3%	8.8%	0.045	8.4%	11.1%	<0.001

ADL, activities of daily living; CCVD, cardio-cerebral vascular disease. The superscripts a and b indicate the difference in the demographics among different subgroups.

The FI of older adults with CCVD was gamma distributed (statistical value = 0.094, *p* < 0.001). The FI of older adults with CCVD was 0.18 (0.11) (ranging from 0.04–0.67) and the FI for women was 0.19 (0.10) higher than the FI for men of 0.17 (0.10) (*z* = 17.495, *p* < 0.001).

**Figure 2:**
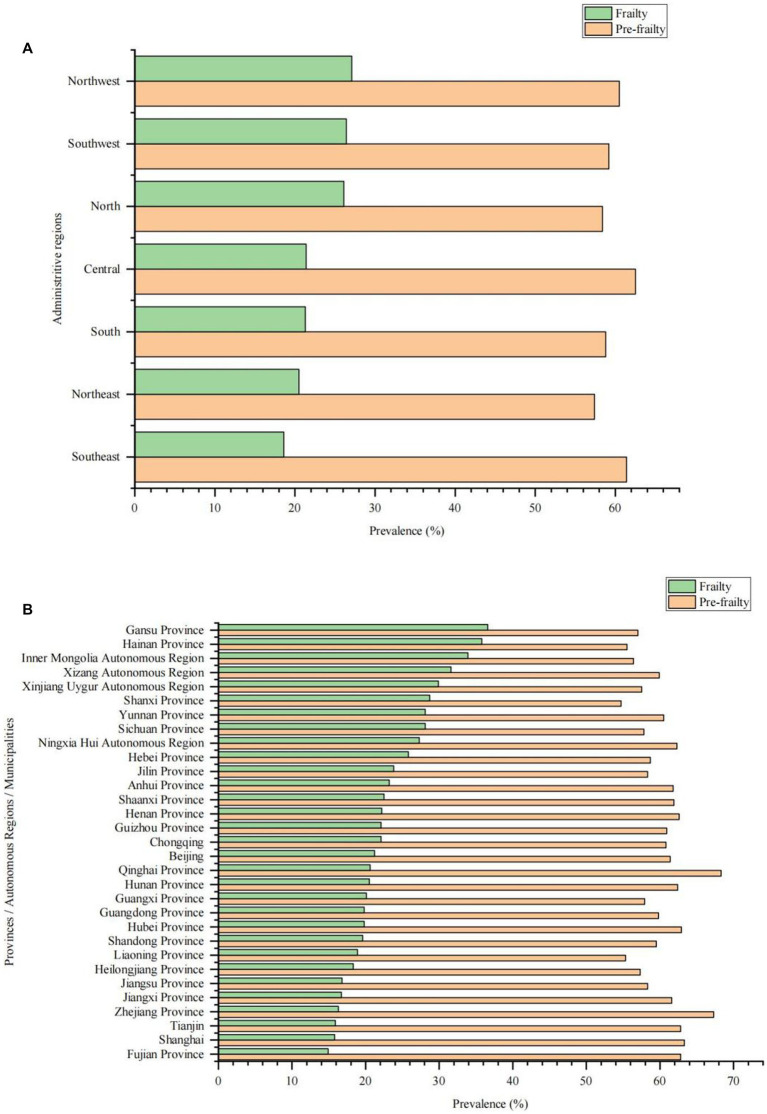
Prevalence of frailty and pre-frailty in older adults with cardio-cerebral vascular disease in different regions in China. **(A)** Prevalence of frailty and pre-frailty in older adults with cardio-cerebral vascular disease in different administrative regions. **(B)** Prevalence of frailty and pre-frailty in older adults with cardio-cerebral vascular disease in different provinces/autonomous regions/municipalities.

The prevalence of frailty in older adults with CCVD increased with age, with a significant increase from 15.5% in the 60–64 age group to 42.1% in the ≥85 age group. Additionally, frailty was more prevalent in rural areas and among ethnic minorities. Frailty was mostly seen in older adults with CCVD who were illiterate, widowed, living alone, had not had a health screening during the last year, had been hospitalized during the last year, had financial difficulties, had difficulties in medical reimbursement, and had combined chronic diseases and ADL disabilities (See Table 2). Furthermore, the prevalence of frailty among older adults with CCVD was higher in northern China (24.0%) than in southern China (20.2%). The prevalence of frailty among older adults with CCVD in seven administrative regions of mainland China was highest in northwest China, followed by southwest China, then north China, then central, south, northeast and southeast China. The highest prevalence of frailty among older adults with CCVD in 31 provinces/municipalities/autonomous regions in mainland China was in Gansu Province (36.6%), while the lowest prevalence was in Fujian Province (14.9%) (Figure 2).

**Table 2:** Prevalence of frailty and pre-frailty among older adults with CCVD in China in 2015.

	Entire population			Men			Women		
	Prevalence of pre-frail	Prevalence of frail	*p* value	Prevalence of pre-frail	Prevalence of frail	*p* value	Prevalence of pre-frail	Prevalence of frail	*p* value
Proportional of participants	60.2%	22.4%		59.7%	20.2%		60.6%	24.0%	
Age group			<0.001			<0.001			<0.001
60–64	61.6%^a^	15.5%^a^		59.5%^a,b^	14.0%^a^		63.1%^a^	16.5%^a^	
65–69	62.1%^a^	18.5%^b^		61.6%^b^	16.3%^b^		62.5%^a,b^	20.2%^b^	
70–74	61.6%^a^	21.4%^c^		61.5%^b^	19.4%^c^		61.6%^a,b^	23.0%^c^	
75–79	60.0%^b^	26.8%^d^		59.9%^a,b^	24.3%^d^		60.0%^b^	28.8%^d^	
80–84	56.4%^c^	32.5%^e^		56.6%^a^	29.9%^e^		56.3%^c^	34.3%^e^	
≥85	49.3%^d^	42.1%^f^		50.6%^c^	38.6%^f^		48.4%^d^	44.3%^f^	
Urban or rural area			<0.001			<0.001			<0.001
Urban	60.7%^a^	20.2%^a^		59.6%^a^	18.7%^a^		61.5%^a^	21.3%^a^	
Rural	59.6%^b^	25.0%^b^		59.7%^a^	22.0%^b^		59.5%^b^	27.3%^b^	
Education			<0.001			<0.001			<0.001
Illiterate	58.5%^a^	29.5%^a^		59.2%^a^	26.4%^a^		58.3%^a^	30.2%^a^	
Primary school	60.7%^b^	21.7%^b^		59.5%^a^	22.0%^b^		61.7%^b^	21.5%^b^	
Junior secondary school	61.2%^b^	16.6%^c^		59.8%^a^	17.1%^c^		63.3%^b^	15.8%^c^	
Senior secondary school	62.0%^b^	15.0%^d^		60.9%^a^	15.2%^c^		63.5%^b^	14.7%^c^	
Tertiary	59.6%^a,b^	14.5%^c,d^		59.3%^a^	13.5%^c^		60.3%^a,b^	16.8%^b,c^	
University or above	62.0%^a,b^	14.1%^c,d^		59.2%^a^	14.7%^c^		68.8%^b^	12.7%^c^	
Marital status			<0.001			<0.001			<0.001
Married	60.5%^a^	18.9%^a^		59.3%^a^	18.3%^a^		61.6%^a^	19.5%^a^	
Widowed	59.0%^b^	31.1%^b^		59.7%^a^	29.9%^b^		58.8%^b^	31.5%^b^	
Divorced	69.2%^c^	19.2%^a,c^		68.5%^b^	20.6%^a,c^		70.0%^a^	17.5%^a^	
Unmarried	67.6%^c^	23.9%^c^		68.2%^b^	23.4%^c^		62.5%^a,b^	29.2%^a,b^	
Ethnicity			<0.001			0.011			0.002
Han	60.2%^a^	22.3%^a^		59.7%^a^	20.0%^a^		60.5%^a^	23.9%^a^	
Ethnic Minorities	60.9%^a^	24.5%^b^		59.1%^a^	23.2%^b^		62.2%^a^	25.5%^a^	
Living status			<0.001			<0.001			<0.001
Living alone	63.4%^a^	31.4%^a^		65.5%^a^	28.3%^a^		62.4%^a^	32.8%^a^	
Not living alone	59.7%^b^	20.9%^b^		59.0%^b^	19.2%^b^		60.2%^b^	22.2%^b^	
Health checkup within 1 year			<0.001			<0.001			<0.001
No	58.5%^a^	24.9%^a^		58.3%^a^	22.3%^a^		58.6%^a^	26.8%^a^	
Yes	61.3%^b^	20.7%^b^		60.5%^b^	18.8%^b^		61.9%^b^	22.2%^b^	
Hospitalized within 1 year			<0.001			<0.001			<0.001
No	61.7%^a^	17.8%^a^		60.6%^a^	15.7%^a^		62.4%^a^	19.4%^a^	
Yes	58.0%^b^	29.1%^b^		58.4%^b^	26.4%^b^		57.8%^b^	31.1%^b^	
Economic status			<0.001			<0.001			<0.001
Very rich	55.0%^a^	14.7%^a^		55.9%^a^	11.7%^a^		54.2%^a,b^	17.4%^a,b^	
Rich	59.5%^b^	16.0%^a^		58.1%^a^	14.1%^a^		60.6%^b,c^	17.4%^b^	
Adequate	61.1%^c^	20.0%^b^		60.2%^a^	18.2%^b^		61.7%^c^	21.4%^a^	
Poor	59.9%^b^	29.5%^c^		60.2%^a^	26.4%^c^		59.6%^b^	31.7%^c^	
Very poor	54.4%^a^	40.7%^d^		56.6%^a^	38.0%^d^		52.7%^a^	42.7%^d^	
Medicare			0.960			0.703			0.575
Yes	60.2%	22.4%		59.7%	20.1%		60.6%	24.0%	
No	60.7%	21.9%		57.1%	22.2%		63.5%	21.6%	
Convenience of medical cost reimbursement			<0.001			<0.001			<0.001
Highly convenient	60.0%^a^	21.4%^a^		59.5%^a^	18.8%^a^		60.4%^a^	23.3%^a^	
Convenient	60.2%^a^	22.0%^a,b^		59.5%^a^	20.0%^a,b^		60.8%^a^	23.5%^a^	
Less convenient	60.9%^a^	22.9%^b^		60.5%^a^	20.9%^a,b^		61.1%^a^	24.3%^a^	
Inconvenient	58.6%^a^	28.2%^c^		58.7%^a^	25.9%^c^		58.5%^a^	30.0%^b^	
Highly inconvenient	60.5%^a^	28.3%^c^		61.0%^a^	25.2%^b,c^		60.0%^a^	30.9%^b^	
Comorbidities			<0.001			<0.001			<0.001
<1	34.5%^a^	5.9%^a^		32.9%^a^	5.9%^a^		36.1%^a^	5.9%^a^	
≥1	64.8%^b^	25.3%^b^		65.4%^b^	23.2%^b^		64.4%^b^	26.8%^b^	
ADL disability			<0.001			<0.001			<0.001
Yes	18.0%^a^	81.6%^a^		17.6%^a^	81.9%^a^		18.2%^a^	81.3%^a^	
No	64.1%^b^	16.8%^b^		63.5%^b^	14.6%^b^		64.7%^b^	18.5%^b^	

ADL, activities of daily living; CCVD, cardio-cerebral vascular disease. The superscripts a, b, c, d, e, and f indicate the difference in the prevalence of pre-frailty and frailty among different groups (adjusted value of *p*s).

Multinomial regression analysis showed that being female, increasing age, living in a rural area, being widowhood, belonging to a ethnic minority, living alone, no having undergone health screening during the last year, experiencing hospitalization during the last year, facing economic hardship, comorbid chronic diseases, experiencing ADL disability were risk factors for frailty and pre-frailty in older adults with CCVD. On the other hand, having received education was a protective factor for frailty and pre-frailty in older adults with CCVD (Table 3).

**Table 3:** Related factors associated with frailty and pre-frailty by multinomial logistic regression of older adults with CCVD.

Variable		Pre-frailty vs. robust				Frailty vs. robust			
		OR	95%CI		*p*-value	OR	95%CI		*p*-value
			Lower	Upper			Lower	Upper	
Sex	Male	1 (ref)							
	Female	1.07	1.008	1.135	0.025	1.147	1.064	1.238	0.001
Age (years)	60–64	1 (ref)							
	65–69	1.146	1.066	1.233	<0.001	1.282	1.164	1.411	<0.001
	70–74	1.293	1.193	1.402	<0.001	1.596	1.438	1.77	<0.001
	75–79	1.636	1.489	1.797	<0.001	2.268	2.019	2.548	<0.001
	80–84	1.905	1.694	2.142	<0.001	3.056	2.656	3.515	<0.001
	≥85	2.296	1.940	2.717	<0.001	4.371	3.604	5.302	<0.001
Urban or rural area	Urban	1 (ref)							
	Rural	1.256	1.183	1.335	<0.001	1.498	1.390	1.615	<0.001
Marriage	Married	1 (ref)							
	Widowed	1.125	1.034	1.224	0.006	1.181	1.067	1.307	0.001
	Divorced	1.237	0.879	1.740	0.223	1.101	0.728	1.667	0.648
	Unmarried	1.414	0.956	2.094	0.083	1.054	0.671	1.656	0.820
Education	Illiterate	1 (ref)							
	Primary school	0.826	0.766	0.891	<0.001	0.757	0.692	0.828	<0.001
	Junior high school	0.709	0.649	0.775	<0.001	0.541	0.484	0.605	<0.001
	Senior high School	0.687	0.614	0.769	<0.001	0.461	0.396	0.536	<0.001
	Tertiary education	0.664	0.556	0.792	<0.001	0.516	0.403	0.661	<0.001
	University or higher	0.624	0.499	0.781	<0.001	0.456	0.333	0.623	<0.001
Ethnicity	Han	1 (ref)							
	Ethnic Minorities	1.232	1.076	1.411	0.002	1.204	1.021	1.420	0.027
Living alone	No	1 (ref)							
	Yes	5.348	4.685	6.104	<0.001	9.332	8.057	10.809	<0.001
Medical checkup within the previous year	Yes	1 (ref)							
	No	1.08	1.019	1.144	0.009	1.212	1.128	1.302	<0.001
Hospitalized within the previous year	No	1 (ref)							
	Yes	1.434	1.352	1.521	<0.001	2.391	2.226	2.569	<0.001
Economic status	Very rich	1 (ref)							
	Rich	1.456	1.179	1.799	0.001	1.476	1.078	2.022	0.015
	Adequate	2.146	1.747	2.636	<0.001	2.838	2.091	3.852	<0.001
	Poor	4.243	3.419	5.266	<0.001	9.164	6.696	12.542	<0.001
	Very poor	10.181	7.582	13.67	<0.001	37.54	25.735	54.76	<0.001
Medical reimbursement	Very convenient	1 (ref)							
	Convenient	1.042	0.979	1.110	0.193	1.041	0.962	1.127	0.317
	Less convenient	1.177	1.084	1.278	<0.001	1.158	1.045	1.283	0.005
	Inconvenient	1.352	1.157	1.580	<0.001	1.71	1.424	2.053	<0.001
	Very inconvenient	1.669	1.314	2.120	<0.001	2.12	1.611	2.79	<0.001
Comorbidities	<1	1 (ref)							
	≥1	16.914	15.851	18.048	<0.001	94.27	82.395	107.858	<0.001
ADL disability	No	1 (ref)							
	Yes	31.886	20.646	49.246	<0.001	696.491	449.316	1079.642	<0.001

CI, confidence interval; OR, odds ratio; ADL, activities of daily living; CCVD, cardio-cerebral vascular disease.

## Discussion

Our large national sample study showed that the prevalence of self-reported CCVD among older adults in China was 25.8%, indicating a very high burden of CCVD among older adults. With the aging of the population, the burden of CCVD has shifted to older adults in the last two decades. The Cardiovascular Health and Diseases Report in China 2021 showed that the prevalence of CCVD in China is in a continuous rise, with about 330 million people currently suffering from CCVD, and the mortality rate of CCVD among Chinese urban and rural residents has also been on the rise in the last decade, with rural and urban CCVD accounting for 46.74 and 44.26% of causes of death, respectively, in 2019 (2). Population aging is the main driver of the increase in deaths from CCVD, with studies showing that 3.09 million residents died from CCVD in China in 2005, increasing to 4.58 million in 2020, representing a 48.06% increase in total mortality from CCVD in China compared to 2005 (20). CCVD has become the most important chronic non-communicable diseases that affect the health and longevity of older adults in China. Frailty is a prevalent age-dependent syndrome that is associated with adverse health outcomes. Studies have shown that CCVD is closely associated with frailty and that frailty has an impact on the immediate and long-term prognosis of patients with CCVD (11, 12). Therefore, studying the frailty status and risk factors of older Chinese patients with CCVD can help inform public health policy makers and promote healthy aging in older patients with CCVD.

Our study used a rigorous sampling design and quality control to accurately report the prevalence of frailty and pre-frailty among Chinese older adults with CCVD. Our study found a high age-standardized prevalence of frailty of 22.6% (95% CI 22.3–23.0%) and an age-standardized prevalence of pre-frailty of 60.1% (95% CI 59.7–60.5%) in older adults with CCVD. Studies have shown that the prevalence of frailty in patients with different degrees of CCVD ranges from 10 to 60%, relying on the definition of frailty and assessment tools used in each study (10, 11). In patients with CCVD, the prevalence of frailty was three times higher than in those without CCVD (21). In our study, the prevalence of frailty in older adults with CCVD was 4.6 times higher than in those without CCVD. Our study found a 61.2% prevalence of CCVD among frail older patients, significantly higher than the 17.4% prevalence of CCVD among robust older adults, and our study shows that cardiovascular disease is strongly associated with frailty in the older adults. Studies have shown a strong bidirectional association between CCVD and frailty (12, 22), with both disease processes having the same pathophysiological basis. Inflammatory senescence, a mild, asymptomatic, systemic, chronic inflammatory state, occurs with age and is characterized by an elevation of pro-inflammatory factors that act as molecular mediators and a decrease in factors that inhibit the inflammatory response. Chronic inflammation plays an important role in the pathophysiology of CCVD, and chronic inflammation also has direct or indirect effects on the musculoskeletal, endocrine, cardiovascular and hematological systems, leading to frailty occurrences (23, 24).

Our study found that that several factors, including being female, increased age, living in a rural area, being widowhood, belonging to a minority, not having undergone health screening during the last year, having been hospitalization during the last year, experiencing financial difficulties, facing inconvenient medical reimbursement, having comorbid chronic diseases, and experiencing ADL disability were risk factors for frailty and pre-frailty in older adults with CCVD, while having received education is a protective factor for frailty and pre-frailty in older adults with CCVD. The findings of our study are consistent with those of previous studies (4, 25–30). The risk factors for frailty in older patients with CCVD identified in our study could help to identify high-risk patients early and implement interventions to prevent or delay the onset of frailty in older patients with CCVD.

Our study found that older women with CCVD had higher rates in the ≥85-year-old age group, were widowed, lived alone, and had comorbid chronic diseases compared with older men with CCVD, Additionally, they had lower rates of good education, health screening during the last year, and financial well-being. These factors may partially explain the gender differences in frailty in older adults with CCVD. Furthermore, our study found that a higher proportion of older adults with CCVD in rural areas were unmarried, ethnic minorities, living alone, in difficult financial situations, experiencing ADL disability, facing inconvenient medical reimbursement, and less well educated compared to older adults with CCVD in urban areas. These factors may partially explain the urban–rural differences in frailty among older adults with CCVD. Additionally, our study found that compared with Han Chinese older adults with CCVD, ethnic minority older adults with CCVD had higher rates of illiteracy, widowhood, past 1-year hospitalization, financial hardship, ADL disability, comorbid chronic diseases, and lower rates of past 1-year health checkups. These differences in demographic characteristics and frailty risk factors may partially explain the ethnic differences in frailty among older adults with CCVD. Lastly, we also found that there were considerable regional differences in the prevalence of frailty among older adults with CCVD, with relatively high prevalence among older adults with CCVD in the less economically developed north-west and south-west regions, and relatively low prevalence in the economically developed coastal regions. These findings may help governments to formulate appropriate policies to prevent and ameliorate the frailty of older adults with CCVD.

Our study has several limitations that should be considered. Firstly, self-reported diagnostic information for CCVD may be subject to recall bias. Secondly, the study lacked information on the specific type, duration and extent of CCVD. Thirdly, smoking is an important risk factor for CCVD and this study did not assess the relationship between smoking and frailty in older patients with CCVD. Lastly, this study was a cross-sectional study and the causative relationship between the factors of interest and frailty in older patients with CCVD could not be determined.

In conclusion, the prevalence of frailty and pre-frailty in older cardiovascular patients is very high and frailty assessment should become routine in the management of older patients with CCVD, attention should be paid to early identification of risk factors for frailty in older patients with CCVD and timely interventions to prevent, delay or reverse the onset of frailty in older patients with CCVD.

## Data availability statement

The original contributions presented in the study are included in the article/supplementary material, further inquiries can be directed to the corresponding author.

## Ethics statement

The studies involving human participants were reviewed and approved by the National Bureau of Statistics (No. [2014] 87) and the Beijing Hospital Ethics Committee (2021BJYYEC-294-01). Written informed consent was provided by all participants. The patients/participants provided their written informed consent to participate in this study.

## Author contributions

X-zZ and NJ wrote the various drafts of the manuscript. X-zZ, L-bM, and JS conducted the statistical analyses. CZ, Y-yL, J-bH, HL, XQ, HW, XH, D-sW, and J-yL participated in data interpretation. D-pL, Q-xZ, JL, and X-zZ conceived and designed this study. X-zZ, NJ, L-bM, CZ, Y-yL, J-bH, JS, HL, XQ, HW, XH, D-sW, J-yL, Q-xZ, JL, and D-pL were drafts of the manuscript for important scientific content. D-pL is the guarantor of this work and, as such, had full access to all the data in the study and takes responsibility for the integrity of the data, and the accuracy of the data analysis. All authors gave final approval of the version to be published.

## Funding

The present study was funded by the National Key R&D Program of China (grant nos. 2020YFC2003000 and 2020YFC2003001). The study sponsors were not involved in the design of the study, the collection, analysis, and interpretation of data, writing the report, or the decision to submit the report for publication.

## Conflict of interest

The authors declare that the research was conducted in the absence of any commercial or financial relationships that could be construed as a potential conflict of interest.

## Publisher’s note

All claims expressed in this article are solely those of the authors and do not necessarily represent those of their affiliated organizations, or those of the publisher, the editors and the reviewers. Any product that may be evaluated in this article, or claim that may be made by its manufacturer, is not guaranteed or endorsed by the publisher.
